# An efficient CRISPR/Cas9 system for simultaneous editing two target sites in *Fortunella hindsii*

**DOI:** 10.1093/hr/uhac064

**Published:** 2022-03-14

**Authors:** Yanhui Xu, Li Zhang, Liqing Lu, Jihong Liu, Hualin Yi, Juxun Wu

**Affiliations:** Key Laboratory of Horticultural Plant Biology, Ministry of Education, Huazhong Agricultural University, Wuhan 430070, China; Key Laboratory of Horticultural Plant Biology, Ministry of Education, Huazhong Agricultural University, Wuhan 430070, China; Key Laboratory of Horticultural Plant Biology, Ministry of Education, Huazhong Agricultural University, Wuhan 430070, China; Key Laboratory of Horticultural Plant Biology, Ministry of Education, Huazhong Agricultural University, Wuhan 430070, China; Key Laboratory of Horticultural Plant Biology, Ministry of Education, Huazhong Agricultural University, Wuhan 430070, China; Key Laboratory of Horticultural Plant Biology, Ministry of Education, Huazhong Agricultural University, Wuhan 430070, China

## Abstract

The CRISPR/Cas9 system is a revolutionary genome editing technique and has been widely used in numerous plants. For plants (e.g. citrus) with very low transformation efficiency, how to optimize gene editing efficiency and induce large-fragment deletion has been the focus of research. Here, we report that CRISPR/Cas9 induces efficient deletion of 16–673 bp fragments in the genome of *Fortunella hindsii*. The ability of two binary vectors, pK7WG2D and pMDC32, to introduce specific mutations into the genome of *F. hindsii* was evaluated. Double single guide RNAs (sgRNAs) were designed to achieve precise editing of two sites of a gene and deletion of fragments between the two sites. The construction of vectors based on Golden Gate assembly and Gateway recombination cloning is simple and efficient. pK7WG2D is more suitable for *F. hindsii* genome editing than the pMDC32 vector. Editing efficiency using the pK7WG2D vector reached 66.7%. Allele mutation frequency was 7.14–100%. Plants with 100% allele mutations accounted for 39.4% (13 100% allele mutation plants/33 mutants). The proportion of mutant plants with fragment deletion induced by this editing system was as high as 52.6% (10 fragment-deletion mutants/19 *FhNZZ* mutants). Altogether, these data suggest that our CRISPR/Cas9 platform is capable of targeted genome editing in citrus and has broad application in research on the citrus functional genome and citrus molecular breeding.

## Introduction

Perennials have to undergo many years of juvenile growth before entering the reproductive stage of flowering and fruiting, which seriously affects the progress of gene function research and breeding of perennial plants. Some short-juvenility germplasms in plants were used as genetic transformation materials to verify the functions of certain genes related to excellent traits, which made it possible to use transgenic technology to improve target characteristics [1, 2]. Due to its versatility and efficiency, CRISPR/Cas9 technology has been widely used in recent years in many annual plant species, including *Arabidopsis thaliana*, tomato, cucumber, rice, and wheat [3–7]. The efficient application of CRISPR/Cas9 gene editing technology in short-juvenility germplasm provides an effective strategy for the discovery of genes controlling excellent traits and the study of gene function.

The CRISPR/Cas9 system has accurate genome editing ability, but editing efficiency and off-target phenomena are still the main constraints. Many optimizations focus on improving editing efficiency and reducing off-target efficiency. The structure of Cas9 and single guide RNA (sgRNA) affect editing efficiency. Codon-optimized *Cas9* gene editing efficiency is significantly improved [8]. The editing efficiency of sgRNA with high GC content (50–70%) guide sequence is relatively higher, and The pairing of guide sequences in sgRNA to form stem-loop structure often reduces the efficiency of genome editing in rice [9]. The editing efficiency of sgRNA with 20 nucleotides guide sequence is the highest, and the editing efficiency of double sgRNAs is much higher than that of a sgRNA [10]. Reducing the length of guide sequence and adding two guanines at the 5′ end of guide sequence can reduce the off-target rate without affecting cutting activity [11, 12]. Use of the sgRNA expression cassette cascade realized the simultaneous knockout of multiple genes [13]. Chemical modification of the 3′- and 5′-terminal three nucleotides of sgRNA improved editing efficiency, and the off-target effect decreased [14]. Environmental factors also affect gene editing efficiency. Heat shock treatment at 37°C increased the directed mutation efficiency of CRISPR/Cas9 in citrus and *A. thaliana* [15]. A CRISPR/Cas9 system designed based on the characteristics of the endogenous tRNA processing system to precisely cut both ends of the tRNA precursor has achieved simultaneous mutation of up to eight target sites in the rice genome, and the efficiency for individual sites has reached 100% [16].

Citrus species are diverse and rich in genetic diversity. The release of genomic information on many citrus species has provided key genetic information to resolve the functions of genes regulating excellent agronomic traits [17–19]. Several attempts have been made to apply CRISPR/Cas9 technology to the functional analysis of citrus genes. However, editing efficiency (11.5–75%) varies widely among different citrus species [18, 20–22]. Although the editing efficiency of the YAO promoter-driven CRISPR/Cas9 system in Carrizo orange reached 75%, the mutation type was mainly single base insertion. Besides, the single sgRNA design may be responsible for 25–87.5% of allele mutations [22]. The CRISPR/Cas9 system with two sgRNAs was successfully applied to *F. hindsii* to produce ~50% editing efficiency [18]. Studies have shown that the combination of sgRNA pairs facilitates microdeletions in the genome [3, 23]. However, the two sgRNAs targeting *F. hindsii* genes were spaced at 400- or 100-bp intervals, while the major mutation type was a 1-bp insertion [18]. Additionally, CRISPR/Cas9-mediated genomic mutation in other perennial plants was mainly characterized by the insertion or deletion of a single base or several bases [24, 25]. The insertion or deletion of a single base or small fragments can easily lead to unpredictable protein products, impeding the study of gene function. Consequently, the development of a CRISPR/Cas9 editing system with the advantages of high editing efficiency, a high frequency of 100% allele mutations, and the ability to mediate large-fragment deletions is essential for functional genomics research in perennial plants.

*F. hindsii* is a wild citrus variety with small crown, short juvenility, and short fruit growth period, and its genetic background is close to that of cultivated citrus [18]. These characteristics make *F. hindsii* an ideal material for the study of citrus genetics and functional genomics. The applicability of the CRISPR/Cas9 system in *F. hindsii* was verified by knocking out the phytic acid dehydrogenase (*PDS*) gene, and ~50% editing efficiency was detected [18]. As a model plant for the study of citrus gene function, the editing efficiency of the CRISPR/Cas9 system in *F. hindsii* needs to be further improved.

In this study, we report a CRISPR/Cas9 system with two sgRNAs mediated by *Agrobacterium tumefaciens*, which demonstrated efficient editing of the *F. hindsii* genome by targeting the MYB transcription factor *DUO POLLEN 1* (*FhDUO1*) and the transcription factor *NOZZLE* (*FhNZZ*) (synonym *SPOROCYTELESS*)*.* Large-fragment efficient deletion was induced by this CRISPR/Cas9 system in *F. hindsii*. Gene editing efficiency and transformation efficiency both reached a high level. The results of this study provide a new CRISPR/Cas9 gene editing tool for citrus functional genomics research and citrus molecular breeding.

## Results

### Efficient and rapid construction of the CRISPR/Cas9 system targeting two sites without PCR

**Figure 1:**
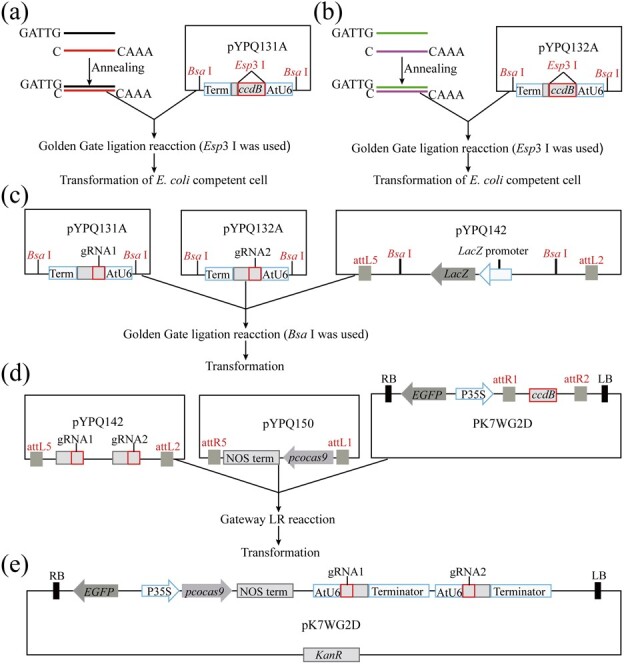
Process of assembly of *Cas9* gene and two sgRNA expression cassettes. **a** and **b** Cloning of a single gRNA into pYPQ131A or pYPQ132A by the Golden Gate reaction and transformation in *E. coli* chemically competent cells. Esp3I is required for digesting pYPQ131A and PYPQ132A. **c** Assembly of two sgRNAs into the pYPQ142 acceptor; BsaI is required. **d** and **e** Assembly of the *Cas9* gene and two sgRNAs into the T-DNA binary vector PK7WG2D (Gateway recombination); P35S initiates *pcoCas9* gene expression, and *kanR* is the kanamycin resistance gene. gRNA1 and gRNA2 represent the guide sequence.

To efficiently construct the CRISPR/Cas9 system, we cloned the toxic gene *ccdB* into the SalI restriction site of the gRNA entry vectors by infusion ligation (Fig. 1a and b). The toxic gene *ccdB* facilitates screening of positive clones after cloning of annealed sgRNA oligonucleotide pairs. The modified vectors pYPQ131A-*ccdB* and pYPQ132A-*ccdB* were workable in cloning sgRNAs with the Golden Gate reaction using the enzyme Esp3I. Two sgRNA transcription units were assembled into the acceptor vector pYPQ142 with a second Golden Gate reaction using the enzyme BsaI (Fig. 1c). LR cloning was performed to assemble the CRISPR/Cas9-related components—the *Cas9* gene and two gRNA expression cassettes—into a binary vector (Fig. 1d and e). Golden Gate ligation and LR cloning were used in the construction of the vector without conventional PCR, plasmid digestion, and postdigestion purification. Furthermore, the system construction can be completed within 10 days (Supplementary Data Fig. S1f). These results showed that the strategy can be fast, simple, and efficient for vector construction, in addition to being friendly for the construction of large-scale dual-target CRISPR/Cas9 systems.

### Different binary T-DNA vectors affect the efficiency of *F. hindsii* gene editing

**Figure 2:**
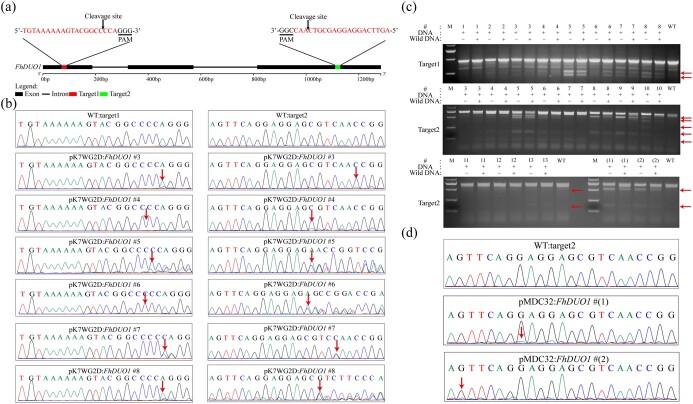
Targeted genomic editing of *FhDUO1* by the CRISPR/Cas9 system. **a** Schematic illustration of the two guide sequences (sequences highlighted in red) targeting the *FhDUO1* coding sequence. **b** Direct sequencing of PCR product chromatograms with overlapping traces demonstrating successful gene editing in the target regions of *FhDUO1* in six representative T0 plants. pK7WG2D:*FhDUO1* #3–8 represent six mutant plants for pK7WG2D:*FhDUO1*. The sequencing chromatograms from wild type (WT) served as the negative control. Red arrows indicate the positions where or from where the mutations occurred. **c** Detection of genomic mutations by T7 endonuclease I (T7E1) assay. The target fragments were amplified by PCR from genomic DNA extracted from transgenic plant leaves. #1–13 represent 13 mutant plants for pK7WG2D:*FhDUO1*. #(1) and #(2) represent two mutant plants for pMDC32:*FhDUO1.* Red arrows point to bands of expected size after T7EI digestion. +, PCR products were added; −, no PCR products were added. **d** Direct sequencing of PCR product chromatograms with overlapping traces demonstrating successful gene editing in the target region of *FhDUO1* in two T0 plants. pMDC32:*FhDUO1* #(1) and #(2) represent two mutant plants for pMDC32:*FhDUO1.* Red arrows indicate the positions where or from where the mutations occurred.

To test whether our CRISPR/Cas9 system can effectively edit the *F. hindsii* genome, we first selected the R2R3-MYB transcription factor *FhDUO1* and designed two target sites in the *FhDUO1* coding region. The two target sites were separated by 1025 bp and spanned two introns (Fig. 2a). Using the above methods, we obtained pMDC32:*FhDUO1* and pK7WG2D:*FhDUO1* recombinant vectors targeting *FhDUO1* to evaluate the effects of different plant genetic transformation binary vectors on *F. hindsii* genetic transformation and gene editing efficiency. A total of 30 T-DNA-inserted T0 plants were obtained from 2522 epicotyl segments for the pMDC32:*FhDUO1* vector. The transformation efficiency was 1.19%. Twenty-one transgenic plants were obtained from 2171 epicotyl segments for the pK7WG2D:*FhDUO1* vector. The transformation efficiency was 0.97% (Table 1). We analyzed the direct sequencing peak map of PCR products containing the target sites. The sequence chromatogram of overlapping traces showed that 14 transgenic plants of the pK7WG2D:*FhDUO1* vector were mutated at target sites, and the pK7WG2D binary vector could effectively edit target genes (Fig. 2b; Supplementary Data Fig. S3). Six of the 14 mutants were simultaneously edited at two target sites (Fig. 2b). However, only two transgenic plants were mutated at one target site for the pMDC32:*FhDUO1* vector, indicating that the editing efficiency of the pMDC32 vector was significantly lower than that of the pK7WG2D vector (Fig. 2d; Supplementary Data Table S4). The gel images of the fragments containing two target sites of *FhDUO1* were amplified and the expected large-fragment deletions were not found between the target sites in 16 pMDC32:*FhDUO1* and pK7WG2D:*FhDUO1* mutant plants (Supplementary Data Fig. S4). Then, we confirmed editing by T7 endonuclease I (T7EI) experiments in 13 mutant plants for pK7WG2D:*FhDUO1* and 2 mutant plants for pMDC32:*FhDUO1*. The results of T7 endonuclease analysis were consistent with the results of direct sequencing of PCR products (Fig. 2c). The editing efficiency of pK7WG2D (66.7%) was significantly higher than that of pMDC32 (6.67%) (Table 1). These results suggested that the pK7WG2D vector is more suitable for *F. hindsii* genome editing than pMDC32.

**Table 1:** Transformation efficiency and editing efficiency of different T-DNA vectors and gRNA GC content.

**Vector**	**Target gene**	**Explant**	**No. of lines**	**Transformation efficiency (%)**	**gRNA**	**No. of lines with mutations**	**Mutation rate (%)**	**gRNA GC content (%)**
2X35S-Cas9-AtU6-sgRNA1-AtU6-sgRNA2-pMDC32	*FhDUO1*	2522	30	1.19	gRNA1	2	6.67	45
					gRNA2			55
CaMV35S-Cas9-AtU6-sgRNA1-AtU6-sgRNA2-pK7WG2D	*FhDUO1*	2171	21	0.97	gRNA1	14	66.70	45
					gRNA2			55
CaMV35S-Cas9-AtU6-sgRNA1-AtU6-sgRNA2-pK7WG2D	*FhNZZ*	1960	51	2.60	gRNA1	19	37.30	55
					gRNA2			65

### Evaluation of large-fragment deletion mediated by CRISPR/Cas9

**Figure 3:**
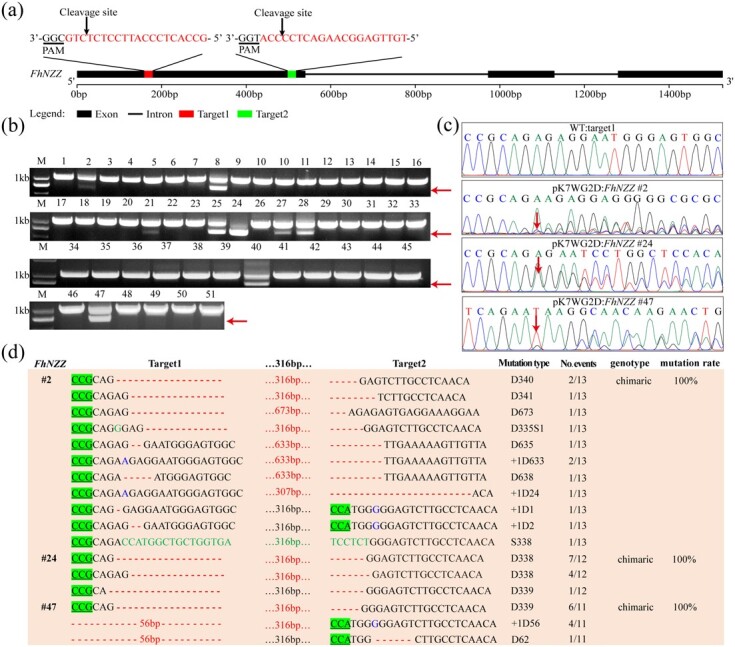
Targeted large-fragment deletions at *FhNZZ* in *F. hindsii*. **a** Schematic illustration of the two guide sequences (sequences highlighted in red) targeting the *FhNZZ* coding sequence. **b** PCR-based detection of deletions at *FhNZZ.* Red arrows indicate the deletion bands. The numbers indicate 51 transgenic plants for pK7WG2D:*FhNZZ.*  **c** Direct sequencing of PCR product chromatograms with overlapping traces demonstrated successful gene editing in the target regions of *FhNZZ* in three representative T0 plants. pK7WG2D:*FhNZZ* #2, #24, and #47 represent three mutant plants for pK7WG2D:*FhNZZ*. The sequencing chromatograms from wild type (WT) served as the negative control. Red arrows indicate the positions where or from where the mutations occurred. **d** Sequence confirmation of different lengths of deletions at *FhNZZ* induced by different sgRNA pairs. #2, #24, and #47 are mutant plants; deletions and insertions are highlighted in red and blue, respectively. Substituted nucleotides are highlighted in green. T-DNA binary vector is pK7WG2D.

Our CRISPR/Cas9 system could mediate short deletion and insertion mutations in *F. hindsii*, which was consistent with previously reported results showing that Cas9/sgRNA mediated small deletion/insertion of target sites in many species [7, 9]. However, CRISPR-Cas9 editors rarely reliably produce substantial deletions at specific targets. To assess whether this system can produce large-fragment deletions in *F. hindsii*, we constructed a pK7WG2D:*FhNZZ* vector targeting *FhNZZ* by adopting different strategies to design sgRNAs. Both target sites of *FhNZZ* are located in the first exon region, separated by 319 bp (Fig. 3a). Fifty-one transgenic plants were screened by fluorescence labeling for pK7WG2D:*FhNZZ*. The transformation efficiency was 2.6% (Table 1). Gel images of the fragment containing two target sites amplified by genomic DNA suggested the large-fragment deletion was present between the target sites (Fig. 3b). The overlapping trace chromatogram of direct sequencing of PCR products shows that 17 of 51 transgenic plants were edited at the *FhNZZ* target sites (Fig. 3c; Supplementary Data Fig. S5). Ten mutant plants contained large fragments with a range of 16–673 bp deletions between two target sites (Fig. 3d; Supplementary Data Table S5). The editing efficiency produced by sgRNAs for *FhNZZ* was 37.3% lower than that of sgRNAs for *FhDUO1* (Table 1; Supplementary Data Table S5). The difference in editing efficiency may arise from the on-target efficiency of the sgRNAs.

### Mutation type

**Figure 4:**
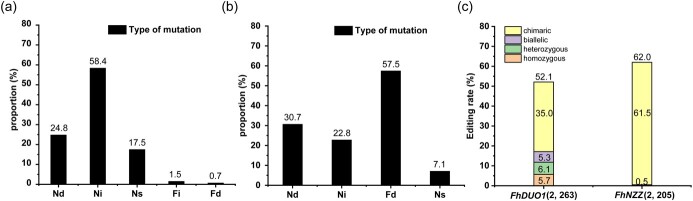
Characterization of targeted editing in *F. hindsii*. **a** Mutation type analysis of the *FhDUO1* gene. The frequency of the different editing events was calculated from the 137 mutation sites. **b** Mutation type analysis of the *FhNZZ* gene. The frequency of the different editing events was calculated from the 127 mutation sites. **c** Editing efficiencies of different allele types driven by the AtU6 promoter in *F. hindsii*. Figures in parentheses are the numbers of involved targets and sequenced sites. Nd, nucleotide deletion; Ni, nucleotide insertion; Ns, nucleotide substitution; Fi, fragment insertion; Fd, fragment deletion.

To study the types of site-specific mutations introduced in *F. hindsii*, we performed monoclonal sequencing of 14 *FhDUO1* mutant plants and 19 *FhNZZ* mutant plants. From the sequencing results, we found that 7.14–100% of alleles had mutations, and the plants with 100% allele mutations accounted for 39.4% (13 100% allele mutation plants/33 mutants) (Supplementary Data Tables S2, S3, and  S5). Among the 263 sequencing sites of plasmid cloning containing a single amplicon for *FhDUO1* targets, 137 (52.1%) mutations were detected, with 15 (5.7%) homozygous mutations, 14 (5.3%) biallelic mutations, 18 (6.1%) heterozygous mutations, and 90 (35.0%) chimeric mutations (Fig. 4c). Among 137 valid sequences, the mutation rate of the target-1 site was 44.9% and that of the target-2 site was 57.1% (Supplementary Data Tables S2 and S3). Four types of targeted mutations were observed in 137 sequences: one nucleotide insertion, one or more nucleotide deletions, fragment deletion, and nucleotide substitutions. The percentage of each mutation type in 137 sequences was calculated (Fig. 4a). The results proved that the mutation types of *FhDUO1* edited by CRISPR are diverse, and they are more inclined to base insertions and deletions.

The results of monoclonal sequencing containing the amplicons for *FhNZZ* targets showed a a high rate (57.5%) of large-fragment deletion mutation (Fig. 4b). In 205 sequencing sites of 19 *FhNZZ* mutant plants, 127 (62%) mutations were detected, with 11 (0.5%) heterozygous mutations and 194 (61.5%) chimeric mutations (Fig. 4c). Our results demonstrated that this CRISPR/Cas9 system is highly efficient at inducing large-fragment deletion.

### Potential off-target analysis

The principal constraints on accurate editing mediated by the Cas9/sgRNA complex are the identification and cutting of non-specific sequences in genomic DNA. The function of other normal genes may be disrupted by altered gene expression patterns when off-target mutations are located in coding regions or non-coding regions with regulatory functions. To investigate whether off-target mutations occur in all 35 mutant plants, we predicted the potential off-target sites of different sgRNAs by using the CRISPR-P 2.0 design tool (http://cbi.hzau.edu.cn/CRISPR2/). Potential off-target sites for *FhDUO1* and *FhNZZ* are shown in Table 2. Direct sequencing chromatograms of PCR products demonstrated off-target mutations in two mutant plants with the pMDC32:*FhDUO1* vector and off-target mutations at one potential off-target site of target 1 (Supplementary Data Fig. S6b). However, 1 of the 14 mutant plants with the pK7WG2D:*FhDUO1* vector exhibited off-target mutations at one potential off-target site of target 2 (Supplementary Data Fig. S6a). The 100% off-target rate of pMDC32 was significantly higher than the 7.14% rate of pK7WG2D. The off-target mutation sites of the two vectors were different. We also estimated five potential off-target mutations for *FhNZZ* by Sanger sequencing of PCR amplification products, and the sequencing chromatogram results indicated that no mutations occurred at these potential off-target sites. The results showed that the mutation rate of potential off-target sites was extremely low in 33 mutant plants for this system based on the pK7WG2D vector.

**Table 2:** Potential off-target sites for two target genes.

**Target**	**Sequence**	**Off-score**	**Number of mismatches**	**Locus**	**Gene**	**Region**
*FhDUO1*-sgRNA1	TATAAGAGAGTACGGGCCCAGGG	0.068	4	chrUn:+46390949	orange1.1 t02992	CDS
*FhDUO1*-sgRNA2	ACTTCAGATGGAGCGTCAGCAGG	0.156	4	chr2:−16750093	Cs2g20030	CDS
*FhNZZ*-sgRNA1	TGTGGAGGAACCACTCCCCACGG	0.113	4	chr2:+7389299	Cs2g10030	CDS
	TTTTGAGGCTAGAATCCACAGGG	0.106	4	chr5:−17741039		Intergenic
*FhNZZ*-sgRNA2	GCCACACCAAGTCCTCTTTGTGG	0.191	4	chr7:−26021287	Cs7g25480	CDS
	GCCACTGCCATTCCACTTTAAGG	0.164	4	chrUn:−4989426	orange1.1 t00332	CDS
					orange1.1 t00333	
	GCCACTCCTTTTCTTCTTTGCAG	0.094	4	chr9:−910208	Cs9g02350	CDS

CDS, coding sequence.

### Mutation in *FhDUO1* affects *F. hindsii* leaf shape and pedicel length

**Figure 5:**
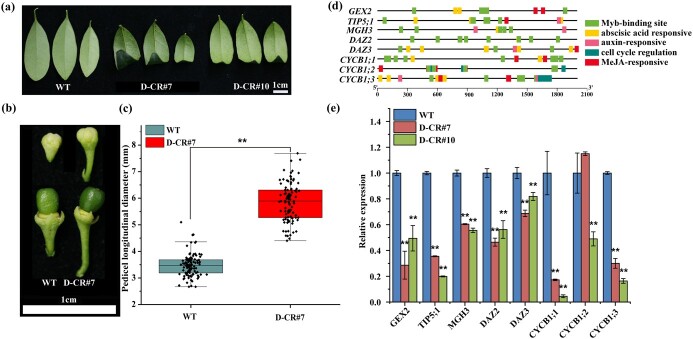
Partial phenotypes of *FhDUO1* mutant plants induced by CRISPR/Cas9. **a** Leaf curling in *duo1* mutant compared with wild type (WT). **b** The *FhDUO1* mutation led to a longer flower peduncle and pedicel at the same developmental stage. **c** Pedicel longitudinal diameter statistics. Values represent mean ± standard deviation (WT, *n* = 100; *FhDUO1* mutants, *n* = 100). **d** Schematic diagram of conserved motifs of downstream target genes of *FhDUO1* elucidated by TBtools [27]. **e** Expression analysis of target genes downstream of *FhDUO1* (mean ± standard deviation, *n* = 3). Asterisks indicate significant differences compared with WT (^**^P < .01, Student’s *t*-test).

In this study, the phenotypes of the *FhDUO1* mutant plants (D-CRs) were e.g. changed, including leaf curling and longer pedicel length (Fig. 5a–c; Supplementary Data Table S6). The leaf curling of D-CR plants was consistent with previous studies [26] (Fig. 5a). In addition, we analyzed the expression of several genes that are the target genes of *FhDUO1* and contain MYB-binding domains in the promoter (Fig. 5d and e). As shown in Fig. 5e, the expression of these genes was significantly downregulated. These results indicated that our CRISPR/Cas9 system successfully changed the encoded protein sequence of *FhDUO1*, and affected the function of *FhDUO1* (Supplementary Data Tables S2 and S3).

## Discussion

In the present study, we developed an efficient and optimized CRISPR/Cas9 system for targeting two genomic loci in *F. hindsii*. We proved that the system can effectively edit the genome of *F. hindsii* and demonstrated the ability of the system to create mutants with large genome fragment deletions in citrus species. The preliminary optimization of this CRISPR/Cas9 system provides a reliable platform for subsequent citrus research.

The application of an efficient vector construction method will be more time-efficient and save effort. Gateway conversion technology and Golden Gate cloning technology have been proven to be efficient cloning systems [28–30]. The sgRNA entry vectors pYPQ131A and pYPQ132A were modified to facilitate the cloning of sgRNAs based on Golden Gate technology (Fig. 1a and b). Two Golden Gate reactions and one LR reaction were performed without PCR, plasmid digestion, and purification of digested products in this system construction (Fig. 1d and e). Therefore, by modifying the sgRNA expression vector, we applied Golden Gate cloning and Gateway recombination to achieve more efficient and rapid vector construction than previously used enzyme analysis and ligation, overlapping PCR, and Gibson assembly [18, 20–22, 31–34]. Moreover, the absence of PCR greatly reduces the probability of base mutation.

*F. hindsii* is a model species for citrus studies and has complete genome sequencing and assembly [18]. However, due to the limitation of genotype dependence, the transformation efficiency of *F. hindsii* is much lower than that of other crops, which increases the workload and further hinders research on citrus genetics and functional genomics [35, 36]. In this study, we confirmed that the CRISPR/Cas9 system with pK7WG2D as the binary vector can efficiently edit the genome of *F. hindsii*, while the pMDC32 vector was not suitable for *F. hindsii* (Figs 2 and 3). As shown in Table 1, there was no significant difference in the transformation efficiency between the two vectors, and both transformation efficiencies were distributed in the normal range (0.2–4%) [18]. However, the editing efficiency of these two vectors is very different. A total of 66.7% of the transgenic plants obtained for pK7WG2D were successfully edited at the target sites, while only 6.67% of the plants obtained for the pMDC32 vector were successfully edited, which is inconsistent with the research results in rice and *Arabidopsis* [37]. Compared with the conventional expression vector, the efficient plant expression vector based on the sweet potato leaf curl virus (SPLCV) replicon can significantly improve the gene editing efficiency of SpCas9 in plants [38]. Two identical sgRNAs were assembled into the pCAMBIA-based vector PHSE-2GR-CHLI and the pMD18T-based vector p2×sgR9-Cas9, and the frequency of mutational events varied considerably [39, 40]. These results showed that the expression vector plays an important role in the editing efficiency of the CRISPR/Cas9 system.

Several CRISPR/Cas9 gene editing systems have been tested in different citrus species [21, 31, 32]. However, the editing efficiency is relatively low, mainly introducing small insertion/deletion (InDel) or substitution mutations at the target loci. The editing efficiency of our CRISPR/Cas9 system based on *F. hindsii* reached 66.7%, which is higher than that of other systems previously published in citrus and higher than the reported system based on *F. hindsii* (50%) [18]. Moreover, a low incidence of off-target mutations was observed. The high editing efficiency may be due to the use of double sgRNA in the system. Designing different sgRNAs helps to optimize targeting efficiency [10, 41]. In addition, the double sgRNA design achieves efficient fragment deletion mutation events between two loci. The proportion of mutant plants with fragment deletion was as high as 52.6% (10 fragment-deletion mutants/19 *FhNZZ* mutants) (Fig. 3b and d; Supplementary Data Table S5). The successful deletion of genomic fragments mediated by the CRISPR/Cas9 system provides a promising strategy for the functional study of small regulatory elements with 5–24 nucleotides, including miRNA, miRNA binding sites and *cis*-acting elements, as well as circRNA molecules that act as miRNA sponges [42, 43].

Through comprehensive evaluation of editing events at four target sites of two genes, the results showed that homozygous and biallelic mutations might be obtained in *F. hindsii* (Supplementary Data Tables S2 and S3). However, the percentage of homozygous and biallelic editing is far lower than that observed in rice [44, 45]. Homozygous mutation is crucial for perennial woody crops, because challenges such as long juvenile period hinder timely evaluation of target phenotypes and delay the speed of crop breeding. At present, the stable genetic transformation of citrus utilizes the epicotyl as explant material, and most of the cells with transformation ability are located among cells undergoing active division on the cambium ring of the explant [46]. A large number of chimeric mutations, even as many as 11 allele mutations, were observed in our research results, which may be attributed to the fact that the mutations occurred after the first cell division of epicotyl transformed cells, and editing continued to occur at the somatic level (Supplementary Data Table S5). It is well known that 100% allele mutations are critical for the study of gene function. Our results showed that this CRISPR/Cas9 system mediated a high proportion (39.4%) of 100% allele mutations, which is considerably higher than that of previous studies [18, 22, 32]. In our *F. hindsii* experiment, the main editing type introduced was single-nucleotide insertion or deletion. In addition, this system can efficiently establish T0 plants with large-fragment deletion mutations in the desired target gene, which made the large-fragment deletion rate of *FhNZZ* mutants up to 57.5% (Fig. 4b; Supplementary Data Table S5). One reason for the large-fragment deletions mediated by our CRISPR/Cas9 system may be that two sgRNAs targeting *FhNZZ* are located in the coding region with an interval of 319 bp. Microhomology-mediated end joining (MMEJ) is another DNA repair mechanism based on recombination between 4- to 25-bp microhomology sequences (MHs) located near double-strand breaks [47]. We identified two groups of 5 bp MHSs (ATGGG) and two groups of 4 bp MHSs (AGTG) in the two sgRNAs of *FhNZZ*, and fragment deletions between the two pairs of MHSs were observed. Therefore, another effective fragment deletion induced by this system may be mediated by MMEJ. Further studies are needed to take these variables into account.

## Conclusions

In summary, we have established an efficient CRISPR/Cas9 editing system in *F. hindsii*. The construction of this system is convenient and efficient. We confirmed that the pK7WG2D vector can achieve efficient editing in *F. hindsii*, and high editing efficiency (66.7%) was obtained in our study. A total of 39.4% of all mutant plants had 100% allele mutations mediated by this CRISPR/Cas9 system. Two sgRNAs with an interval of 319 bp were designed for large-fragment deletion, and the proportion of mutant plants with fragment deletions was as high as 52.6% in this study. The efficient application of this editing system will expedite functional genomics research on citrus and provide a powerful tool for future perennial plant breeding and improvement.

## Materials and methods

### Plant material

*F. hindsii* planted in the National citrus Breeding Center at Huazhong Agriculture University was used as the genetic transformation material. The seeds of *F. hindsii* were collected from ripe fruits. Seedling epicotyls of these seeds were used for genetic transformation.

### Construction of CRISPR/Cas9 vectors

The system assembled *Cas9* and two sgRNAs into T-DNA vectors for targeted editing at two different sites. The protocol for vector construction was conducted as previously described and some steps were modified to achieve more efficient construction [37]. The CRISPR/Cas9 system includes four entry vectors and a T-DNA binary vector (Supplementary Data Fig. S1). These vectors were purchased from Addgene (https://www.addgene.org/). pYPQ131A and pYPQ132A are sgRNA entry vectors containing sgRNA expression cassettes. BsaI restriction enzyme recognition sites on both sides of the sgRNA expression cassette facilitate Golden Gate entry assembly. To efficiently screen monoclonals containing successful cloning plasmids, we inserted the toxic gene *ccdB* into the SalI restriction sites by homologous recombination (Supplementary Data Fig. S1a and b). pYPQ142 is a Golden Gate receptor vector for assembling two sgRNA expression cassettes. The LR sites attL5 and attL2 on both sides of the BsaI restriction sites make it a Gateway entry vector (Supplementary Data Fig. S1c). pYPQ150 is a Cas9 entry vector containing the plant codon optimized *Cas9* (*pcoCas9*) gene without a promoter. *Cas9* flanks the attL1 and attR5 recombination sites (Supplementary Data Fig. S1d). Two T-DNA binary vectors, pK7WG2D and pMDC32, with attR1 and attR2 recombination sites, were used in this study (Supplementary Data Fig. S1e and Fig. S2). pK7WG2D and pMDC32 are commonly used Gateway vectors for *Agrobacterium*-mediated plant genetic transformation.

Four steps are required for vector construction. The first step is the annealing of single-stranded oligonucleotides (Supplementary Data Table S1). The annealing reaction system was as follows: gRNA-oligo forward (10 μM) 19 μl, gRNA-oligo reverse (10 μM) 19 μl, Buffer G (Thermo Scientific) 2 μl; the reaction was precisely annealed at 55°C for 25 minutes (Fig. 1a and b). The second step is to clone annealed oligonucleotide pairs into sgRNA entry vectors by the Golden Gate reaction to ensure a complete sgRNA expression cassette under the *A. thaliana* U6 (AtU6) promoter. The IIS class restriction enzyme *Esp*3 I is required in the ligation step. The Golden Gate reaction system was prepared on ice as follows: Esp3I (Thermo Scientific) 1 μl, T4 DNA ligase (400 U/μl, NEB) 1 μl, 10 mM ATP 2 μl, Buffer G (Thermo Scientific) 2 μl, pYPQ131A or pYPQ132A (100 ng/μl) 0.7 μl, annealed oligonucleotide products 1 μl, sterile distilled water up to 20 μl. The Golden Gate reaction program was run in a thermocycler as follows: 10 cycles for 5 minutes at 37°C and 10 minutes at 16°C, then 10 minutes at 50°C, 20 minutes at 80°C, and 10°C hold. The ligated products were transformed into 50 μl of DH5α competent cells, coated on LB solid plates containing tetracycline (5 ng/μl), and cultured overnight at 37°C (Fig. 1a and b). Two single colonies were selected for PCR verification, and then the positive bacterial solution was verified by Sanger sequencing of sgRNA with 131-F primers. The third step is to assemble two sgRNA expression cassettes into the pYPQ142 vector by the Golden Gate reaction. The following reagents were added in a microcentrifuge tube on ice: 10× T4 DNA ligase buffer (NEB) 1 μl, pYPQ142 (100 ng/μl) 0.6 μl, pYPQ131A-gRNA1 (100 ng/μl) 1.4 μl, pYPQ132A-gRNA2 (100 ng/μl) 1.4 μl, BsaI (Thermo Fisher) 1 μl, T4 DNA ligase (400 U/μl, NEB) 1 μl, sterile distilled water 12.3 μl. The cycle conditions were as follows: 10 cycles for 5 minutes at 37°C and 10 minutes at 16°C, 10 minutes at 50°C, 5 minutes at 80°C, and 10°C hold. The ligation products were transformed into chemically competent *Escherichia coli* DH5α cells, spread on LB solid plates containing spectinomycin (100 μg/ml) and incubated at 37°C overnight. Blue-and-white screening was performed because the sgRNA expression cassette replaced the *LacZ* gene in the pYPQ142 receptor vector (Fig. 1c). Two clones were selected for PCR verification with gene-gRNA-oligo-1F and gene-gRNA-oligo-2R primers, and the clones were further verified by Sanger sequencing. The fourth step is to assemble the *Cas9* gene and sgRNA expression cassettes into the T-DNA binary vector pK7WG2D or pMDC32 by Gateway recombination. Preparation of the 10 μl LR reaction was as follows: pYPQ150 (100 ng/μl) 1.4 μl, pYPQ142-gRNA1-gRNA2 (100 ng/μl) 0.8 μl, T-DNA binary vector (100 ng/μl) 2.4 μl, LR Clonase II enzyme (Invitrogen) 0.5 μl, and sterile distilled water 4.9 μl. The reaction was performed overnight at 25°C in a dry thermostat. All ligation mixtures were transformed into chemically competent *E. coli* DH5α cells directly without terminating the reaction, spread on LB solid plates containing spectinomycin (100 μg/ml), and then incubated at 37°C overnight (Fig. 1d). Ten clones were selected for PCR verification with gRNA-oligo-1F and gRNA-oligo-2R primers, and the sgRNAs and *Cas9* fragments were further verified by Sanger sequencing with gene-gRNA-oligo-1F, gene-gRNA-oligo-2R, and Cas9-F primers. This binary recombinant plasmid was introduced into the chemoreceptor cells of *A. tumefaciens* EHA105 by chemical transformation (Fig. 1e). The concentration of each plasmid in the ligation system was calculated with the formula: plasmid size × 16.5/plasmid concentration. Plasmid size is expressed in kilobases; for example, the pYPQ131A size is ~3.8 kb, and if the plasmid concentration is 100 ng/μl, the volume of the added plasmids is (3.8 × 16.5/100) μl. The added amount of each plasmid was accurately calculated by the formula, which solved the problem that pYPQ142 and pK7WG2D had the same bacterial resistance, and improved the efficiency of vector construction. The primers used in the CRISPR experiment are shown in Supplementary Data Table S7.

### Design of sgRNA

Specific 20-bp-length guide sequences were designed and screened using the CRISPR-P2.0 design tool (http://cbi.hzau.edu.cn/CRISPR2/). According to the characteristics of gene expression, the target site located in the gene’s ORF (open reading frame) is more likely to result in the loss of gene function. Two guide sequences located in the exon regions of the target gene were selected based on the principle of high on-target efficiency score and a low score of sgRNA off-target potential. GATT and AAAC were added to the 5′ ends of forward and reverse oligonucleotides, which are complementary to the four-base sticky ends generated by Esp3I digestion. Transcription of sgRNAs (target sgRNAs) of fusion target sequences initiated by U6 or U3 promoters has definite transcription start sites. The U6 and U3 promoters correspond to guanine and adenine nucleotides, respectively. An adenine was added in front of the 20-bp guide sequence to ensure that the structure of the sgRNA was G(N)_20_. Adding a G before sgRNA does not affect the ability to guide common Cas9 to recognize the CRISPR sequence, and expand the screening range of genomic sgRNA target sequences. The final form of the forward oligonucleotide was GATTG (N)_20_, and the reverse was AAAC (N)_20_C.

### *Agrobacterium*-mediated genetic transformation

*A. tumefaciens* EHA105 mediated the genetic transformation of *F. hindsii*. The steps of genetic transformation were performed according to the previous description [18].

### T7 endonuclease I assay and genotyping

Genomic DNA was extracted from *F. hindsii* leaves, and specific PCR primers were designed to amplify fragments with sgRNA targeting sites. The PCR products were directly sequenced. The heterozygosity and biallelic mutations were preliminarily judged according to the superimposed sequencing chromatograms of direct sequencing results of PCR products. To further observe the different types of mutations in T0 plants, the PCR products were cloned into zero background pTOPO-Blunt Simple vector (Aidlab, China). The primer sequences used are listed in Supplementary Data Table S7.

For mutation detection based on T7EI, the target fragment with mutation sites was amplified by PCR to avoid the target sites in the middle of the target fragment. Ten microliters of the PCR reaction products were added together with 2 μl of 10× NEB Buffer 2 to a PCR tube and mixed gently and then placed into a thermal cycler for denaturation and annealing as follows: initial denaturation at 95°C for 5 minutes, ramp down to 85°C at −2°C/second, then ramp down to 25°C at −0.1°C/second, and 4°C for 5 minutes. Then, 1 μl of T7EI (NEB) was added to the annealed PCR product and incubated for 5 minutes at 37°C to inactivate the T7EI.

### Off-target analysis

To detect off-target events, potential genome-wide off-target sites were predicted using the CRISPR-P 2.0 design tool (http://cbi.hzau.edu.cn/CRISPR2/). Potential off-target sites (off-score >.06 as criterion) are shown in Table 2. Genomic DNA was extracted from *F. hindsii* leaves, and then the putative off-target sites were amplified by PCR with specific primers (Supplementary Data Table S7). The PCR products were directly sequenced for sequencing chromatogram analysis and cloned into the pTOPO-Blunt Simple vector for monoclonal analysis.

### Quantitative PCR analysis

Total RNA was extracted from leaves of *F. hindsii* by the Trizol method. Reverse transcription was performed using the Maxima H Minus First Strand cDNA Synthesis Kit with dsDNase (Thermo Scientific, USA), and quantitative PCR was performed according to the methods of our previous study [48]. *CsACTIN* was used as the endogenous reference gene [49]. The primers used in the experiment are shown in Supplementary Data Table S8.

## Supplementary Material

Web_Material_uhac064

## Data Availability

All relevant data are presented within the paper and its supplementary materials. The raw data that support the findings of this study are available from the corresponding author upon reasonable request.
