# Supplementary material for: An efficient CRISPR/Cas9 system for simultaneous editing two target sites in Fortunella hindsii
Source: Hortic Res. 2022 Mar 14;9:uhac064. doi: 10.1093/hr/uhac064 (PMC9166532; doi:10.1093/hr/uhac064)
Supplement: Web_Material_uhac064 [file web_material_uhac064.docx]

**Supplementary information**

**Figure S1. A CISPR/Cas9 platform for *F. hindsii* plants.**

**Figure S2. Structure of the T-DNA binary vector pMDC32.**

**Figure S3.** **Targeted genomic editing of *FhDUO1* by the CRISPR/Cas9 system.**

**Figure S4. Gel image of *FhDUO1* fragments amplified from genomic DNA*.***

**Figure S5. Targeted genomic editing of *FhNZZ* by the CRISPR/Cas9 system.**

**Figure S6. Off-target editing of sgRNAs by the CRISPR/Cas9 system** **using different binary vector.**

**Table S1. Oligonucleotide sequences used for Golden Gate cloning of sgRNAs.**

**Table S2. Targeted genomic mutation of *FhDUO1* target-1 in T0 plants of *F. hindsii*.**

**Table S3. Targeted genomic mutations of *FhDUO1* target-2 in T0 plants of *F. hindsii*.**

**Table S4. Targeted genomic mutations in T0 plants of *F. hindsii*.**

**Table S5. Targeted genomic mutations of *FhNZZ* in T0 plants of *F. hindsii*.**

**Table S6. Raw statistics of the pedicel longitudinal diameter.**

**Table S7. The primers used in the CRISPR experiment.**

**Table S8. The primers used for expression analysis.**


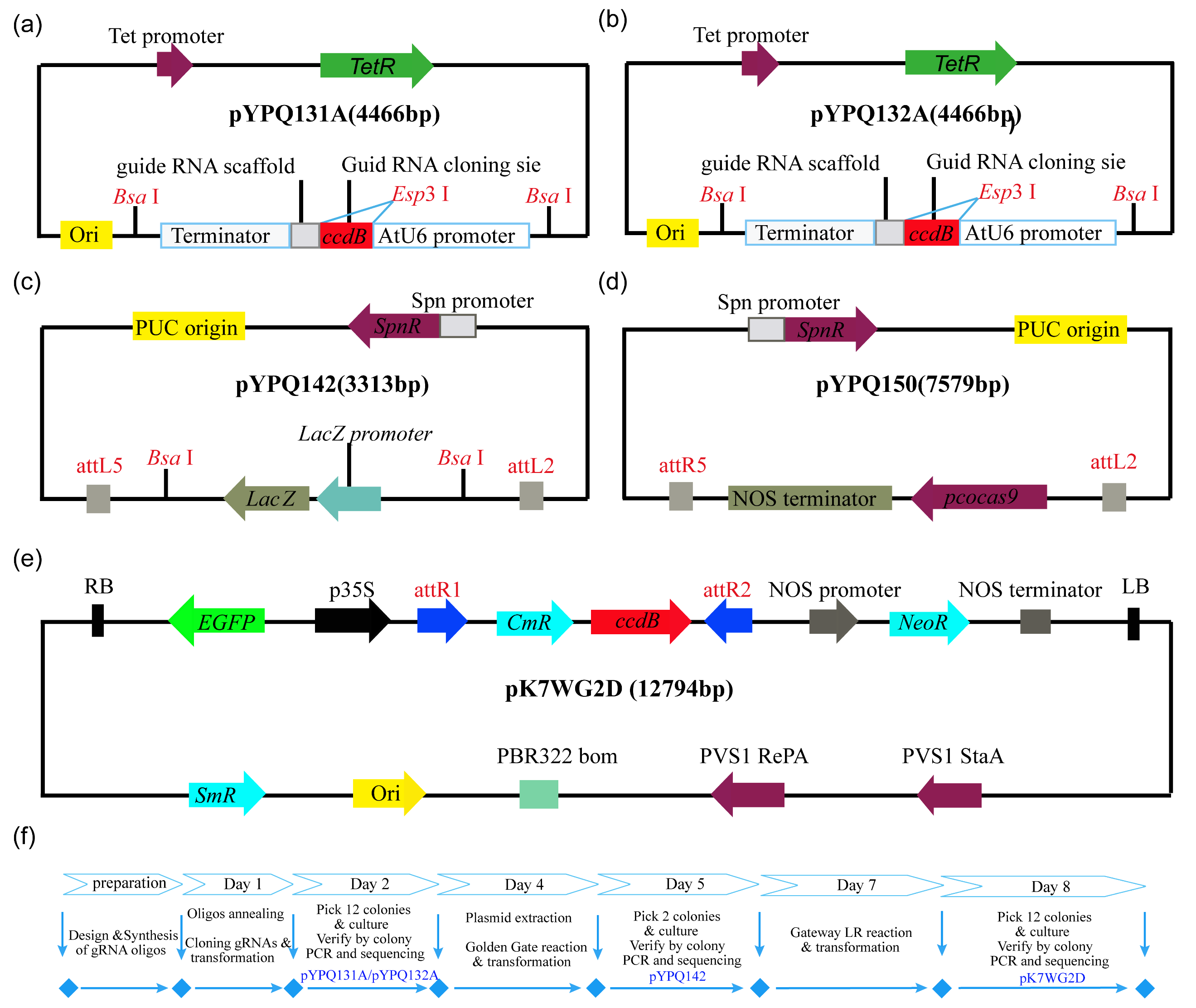


**Figure S1. A CISPR/Cas9 platform for *F. hindsii* plants.** (a) and (b) Structure of the gRNA cloning vector, which is a compatible Golden Gate entry vector. The gRNA promoter is AtU6. (c) Structure of the Golden Gate recipient vector pYPQ142 (for two gRNA expression cassettes), which contains LR sites attL5 and attL2. (d) Structure of the *Cas9* entry vector pYPQ150, attR5 and attL1 for performing the LR reaction. (e) Structure of the T-DNA binary vector pK7WG2D, EGFP, fluorescence reporter gene. P35S, CaMV35S promoter. attR1 and attR2 are used to recombine the *Cas9* gene and sgRNA cassettes into the binary vector pk7WG2D. (f) Timeline for the detailed workflow of vector construction.


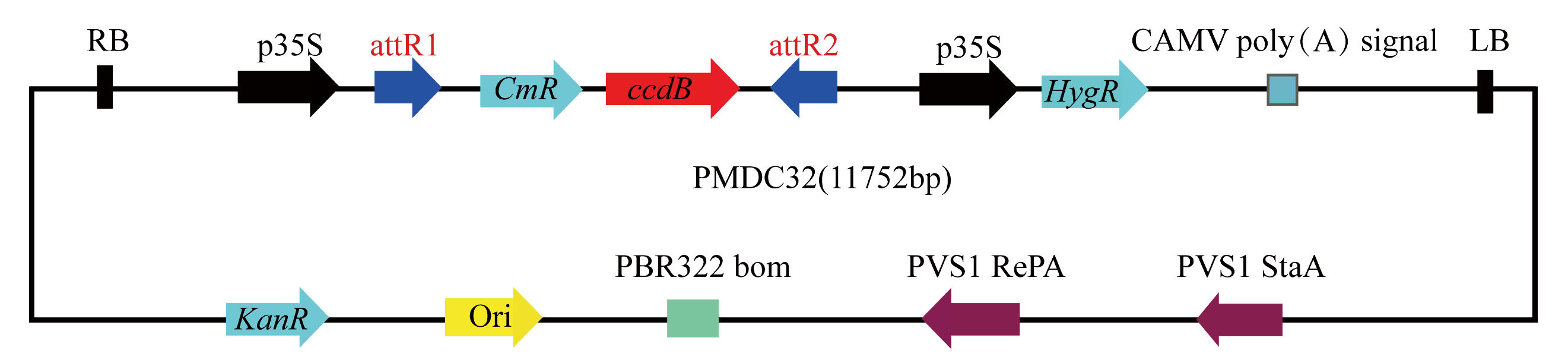


**Figure S2. Structure of the T-DNA binary vector pMDC32.** P35S, CaMV35S promoter; attR1 and attR2 are used to recombine *Cas9* gene and sgRNAs cassettes into the binary vector pMDC32, *kanR* is the kanamycin resistance gene.


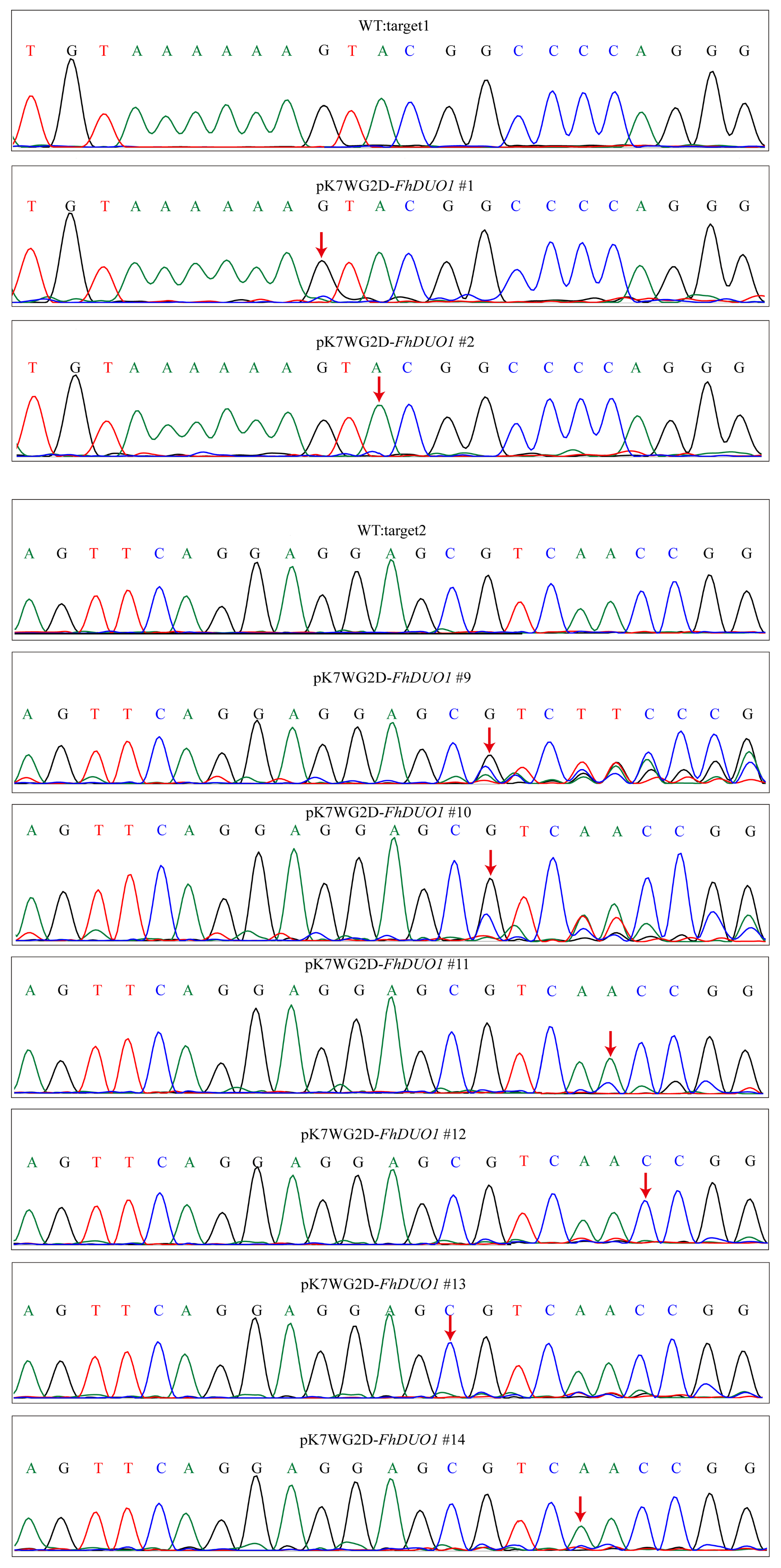


**Figure S3.** **Targeted genomic editing of *FhDUO1* by the CRISPR/Cas9 system.** Direct sequencing of PCR products chromatograms with overlapping traces demonstrating successful gene editing in the target regions of *FhDUO1* in 8 representative T0 plants. pK7WG2D-*FhDUO1* #1-14 represent 8 mutant plants for pK7WG2D: *FhDUO1*.


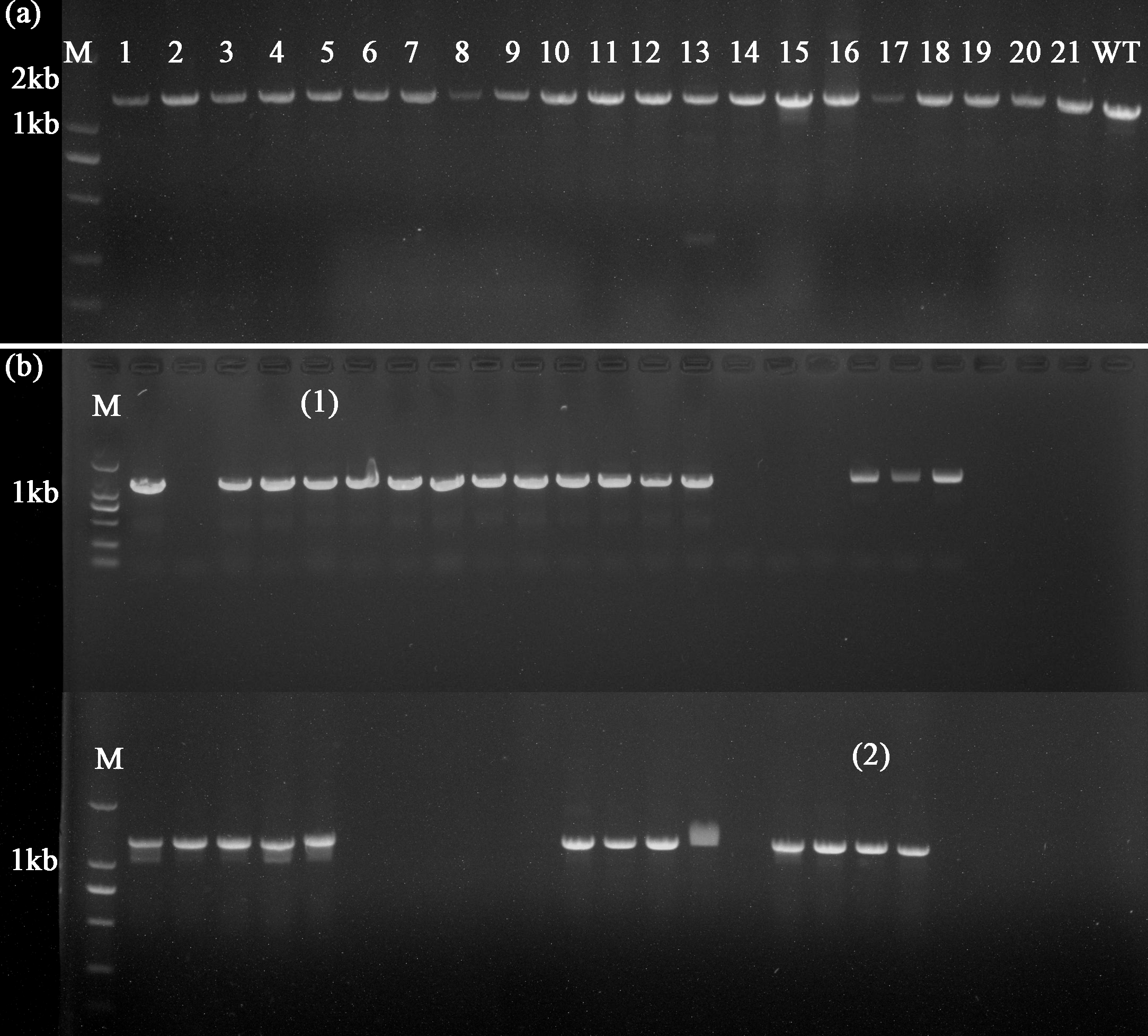


**Figure S4.** **Gel image of *FhDUO1* fragments amplified from genomic DNA*.*** #1-21 represents 21 transgenic plants for pK7WG2D:*FhDUO1.* #(1)-(2) represents 2 transgenic plants for pMDC32:*FhDUO1.*


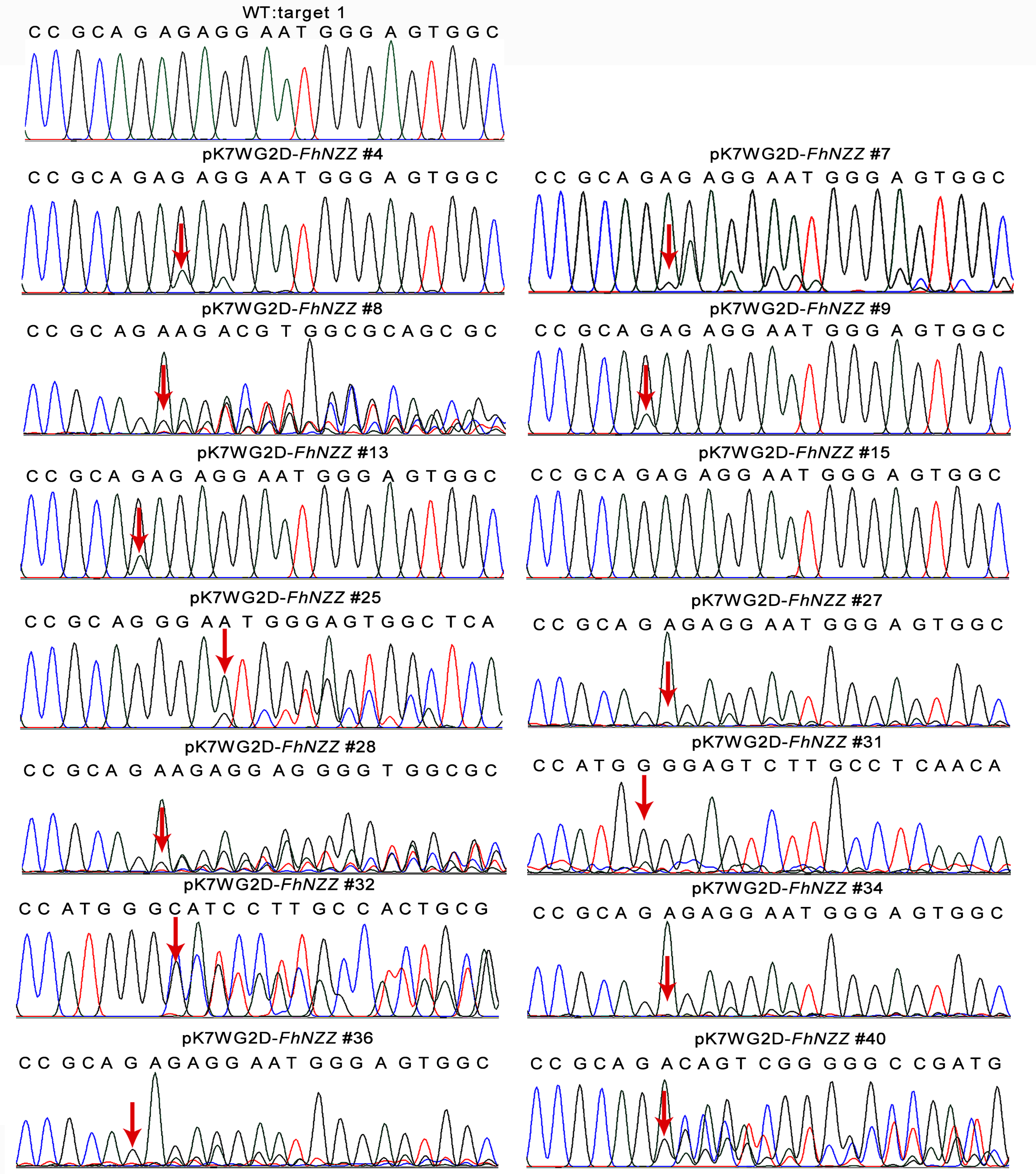


**Figure S5. Targeted genomic editing of *FhNZZ* by the CRISPR/Cas9 system.** Direct sequencing of PCR products chromatograms with overlapping traces demonstrating successful gene editing in the target regions of *FhNZZ* in 14 T0 plants. pK7WG2D-*FhNZZ* #4-40 represent 14 mutant plants for pK7WG2D: *FhNZZ*.


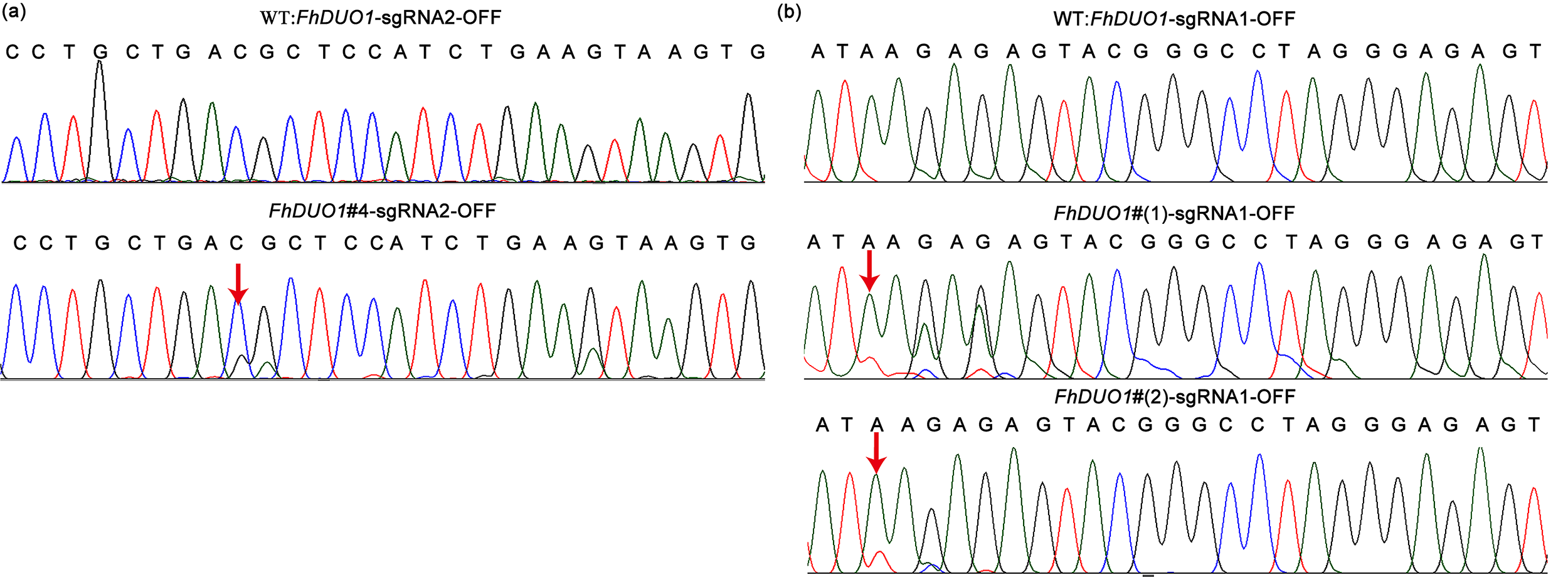


**Figure S6. Off-target editing of sgRNAs by the CRISPR/Cas9 system** **using different binary vector.** Direct sequencing of PCR products chromatograms with overlapping traces demonstrating off-target effects. (a) The binary vector is pK7WG2D. (b) The binary vector is pMDC32.

| **Table S1. Oligonucleotide sequences used for Golden Gate cloning of sgRNAs.** | | | |
| --- | --- | --- | --- |
| Gene name | Gene No. | oligonucleotides name | Sequences (5’-3’) |
| *FhDUO1* | Cs1g03470.1 | DUO1-gRNA-oligo-1F | GATTGTGTAAAAAAGTACGGCCCCA |
|  |  | DUO1-gRNA-oligo-1R | AAACTGGGGCCGTACTTTTTTACAC |
|  |  | DUO1-gRNA-oligo-2F | GATTGAGTTCAGGAGGAGCGTCAAC |
|  |  | DUO1-gRNA-oligo-2R | AAACGTTGACGCTCCTCCTGAACTC |
| *FhNZZ* | Cs1g06080.1 | NZZ-gRNA-oligo-1F | GATTGTGTTGAGGCAAGACTCCCCA |
|  |  | NZZ-gRNA-oligo-1R | AAACTGGGGAGTCTTGCCTCAACAC |
|  |  | NZZ-gRNA-oligo-2F | GATTGGCCACTCCCATTCCTCTCTG |
|  |  | NZZ-gRNA-oligo-2R | AAACCAGAGAGGAATGGGAGTGGCC |

| **Table S2. Targeted genomic mutation of *FhDUO1* target-1 in T0 plants of *F. hindsii*.** #1-#8 are mutant plants for pK7WG2D:*FhDUO1*. The PAM is highlighted with a green background. Deleted nucleotides are highlighted in red. Inserted nucleotides are highlighted in blue. Replaced nucleotides are highlighted in green. plus (+) signs, letter D and letter S indicate the number of nucleotides inserted, deleted and replaced, respectively. The asterisks indicate the numbers of independent clones sequenced. | | | | | |
| --- | --- | --- | --- | --- | --- |
| *FhDUO1* | Target-1 | Mutation detected | No. mutated /No. sequenced | genotype | mutation rate |
| #1 | TGCTTCATCTTCTTCTGCTTTCC  TGTAAAAAAGTACGGCCCCAGGG | S15  WT | 1/14 | heterozygous | 7.14% |
| #2 | TGTAAAAAAGTACGGCCTCAGGG  TGCTTCATCTTCTTCTGCTTTCC  TGTAAAAAAGTACGGCCCCAGGG | S1  S15  WT | 1/15  1/15 | chimeric | 13.33% |
| #3 | TGTAAAAAAGTACGGCCCCCAGGG  TGTAAAAAAATACAGCCCCAAGG  TGTAAAAAAGTACGGCCCCAGGG | +1  S3  WT | 1/15  1/15 | chimeric | 13.33% |
| #4 | TGTAAAAAAGTACGGCCCCCAGGG  CGTAAAAAAGTACGGCCCCAGGG  TGTAAAAAAGTACGGCCCCAGGG | +1  S1  WT | 1/14  1/14 | chimeric | 14.29% |
| #5 | TGTAAAAAAGTACGGCC - CAGGG  TGTAAAAAAGTACGGCCCCCAGGG  GCTAAAAAAGTTCGGCCCCCAGGG  CGTAAAAAAGTGCGGCCCCCAGGG  GAAGAAAAAGTGCGACCCCCA GGG  GGTAAAAAGAGCTAGGAAGATGAGCAGAGATTAGTAAAGGGCCAGGG  AGTAAGAAGAGATAAGAAGAAGTCAAGTGATAAGGAACGGCCCCAGGG | D1  +1  +1S3  +1S2  +1S8  +24S6  +25S4 | 4/14  5/14  1/14  1/14  1/14  1/14  1/14 | chimeric | 100% |
| #6 | TGTAAAAAAGTACGGCCCCCAGGG | +1 | 15/15 | homozygous | 100% |
| #7 | TGTAAAAAAGTACGGCCCCCAGGG  TGTAAAAAAGTACGGCCCCAGGG | +1  WT | 9/15 | heterozygous | 60% |
| #8 | TGTAAAAAAGTACGGCCCCCAGGG  TGTAAAAAAGTACGGCCACCAGGG   Nucleotide substitution  TGTAAAAAAGTACGGCCCCAGGG | +1  +1  +11S12  WT | 1/15  1/15  1/15 | chimeric | 20% |
|  |  |  | 48/107 |  | 44.9% |

| **Table S3. Targeted genomic mutations of *FhDUO1* target-2 in T0 plants of *F. hindsii*.**  #3-#14 are mutant plants for pK7WG2D:*FhDUO1*. The PAM is highlighted with a green background. Deleted nucleotides are highlighted in red. Inserted nucleotides are highlighted in blue. Replaced nucleotides are highlighted in green. plus (+) signs, letter D and letter S indicate the number of nucleotides inserted, deleted and replaced, respectively. The asterisks indicate the numbers of independent clones sequenced. | | | | | |
| --- | --- | --- | --- | --- | --- |
| *FhDUO1* | Target-2 | Mutation detected | No. mutated /No. sequenced | Genotype | Mutation rate |
| #3 | AGTTCAGGAGGAGCGTCAACCGA AGTTCAGGAGGAGCGTCAACCGG | S1  WT | 1/14 | heterozygous | 7.14% |
| #4 | AGTTCAGGAGGAGCGTCAAACCGG  AGTTCAGGAGGAGCGTCAACCGG | +1  WT | 3/15 | heterozygous | 20% |
| #5 | AGTTCAGGAGGAG - - - - AACCGG  AGTTCAGGAGGAGCGTCGGTGAAAAAGTACCGG  AGTTCAGGAGGAGCGTCAACCGG | D4  +10S1  WT | 8/14  2/14 | chimeric | 71.43% |
| #6 | AGTTCAGGAGGAG - - - - - AACCGG  AGTTCAGGAGGAGCGTCAAACCGG  AGTTCAGGAGGAGCGTCAACCGG | D5  +1  WT | 6/14  6/14 | chimeric | 85.71% |
| #7 | AGTTCAGGAGGAGCGTCCAACCGG  AGTTCAGGAGGAGCGTCTAACCGG | +1  +1 | 4/14  10/14 | biallelic | 100% |
| #8 | AGTTCAGGAGGAGCGTCAAACCGG  AGTTCAGGAGGAGCGT A - ACCGG  AGTTCAGGAGGAGCGTCTAACCGG  AGTTCAGGAGGAG - - - - AACCGG | +1  D1  +1  D4 | 7/14  1/14  4/14  1/14 | chimeric | 100% |
| #9 | AGTTCAGGAGGAGCGTCAAACCG  AGTTCAGGAGGAGCG - - AACCGG  AGTTCAGGAGGAGCGTCTAACCGG  AGTTCAGGAGGAG - - - - AACCGG  AGTTCAGGAGGAC - - - - - ACCGG  AGTTCAGGAGGAGCGTCGAACCGG  AGTTCAGGAGGAGC - - - AACCGG  AGTTCAGGAGGAGCGTC - ACCGG  AGTTCAGGAGGA - - - - CAACCGG  AAATGGCTGTCCG---30bp del---AACACA | +1  D2  +1  D4  D5S1  +1  D3  D1  D4  D30 | 2/14  1/14  2/14  2/14  1/14  1/14  2/14  1/14  1/14  1/14 | chimeric | 100% |
| #10 | AGTTCAGGAGGAGC - - - - ACCGG  AGTTCAGGAGGAG - - - - AACCGG  Nucleotide substitution  AGTTCAGGAGGAGCGTCAACCGG | D4  D4  S1  WT | 1 /14  1/14  7/14 | chimeric | 64.29% |
| #11 | AGTTCAGGAGGAGCGTCA - CCGG  AGTTCAGGAGGAGCGTCTAACCGG  AGTTCAGGAGGAGCGTCAAACCGG  AGTTCAGGAGGAGCGTCAACCGG | D1  +1  +1  WT | 2/15  2/15  1/15 | chimeric | 33.33% |
| #12 | AGTTCAGGAGGAGCGTCAAACCGG  AGTTCAGGAGGAGCGTCAACCGG | +1  WT | 1/15 | heterozygous | 6.67% |
| #13 | AGTTCAGGAGGAGC - - - AACCGG  AGTTCAGGAGGAGA - - - AACCGG  AGTTCAGGAGGAGCGTCTACCGG  Nucleotide substitution  AGTTCAGGAGGAGCGTCAACCGG | D3  D3S1  +1  +4S105  WT | 1/15  1/15  1/15  2/15  10/15 | chimeric | 33.33% |
| #14 | AGTTCAGGAGGAGCGTCCAACGG  AGTTCAGGAGGAGCGTCAACCGG | +1  WT | 1/13  12/13 | heterozygous | 7.69% |
|  |  |  | 89/156 |  | 57.1% |

| **Table S4. Targeted genomic mutations in T0 plants of *F. hindsii*.** #(1)-#(2) are mutant plants for pMDC32:*FhDUO1*. The PAM is highlighted with a green background. Deleted nucleotides are highlighted in red. Inserted nucleotides are highlighted in blue. Replaced nucleotides are highlighted in green. plus (+) signs, letter D and letter S indicate the number of nucleotides inserted, deleted and replaced, respectively. The asterisks indicate the numbers of independent clones sequenced. | | | | | |
| --- | --- | --- | --- | --- | --- |
| *FhDUO1* | Target-2 | Mutation detected | No. mutated/No. sequenced | genotype | mutation rate |
| # (1) | AGACCCTTTTT - - CGCTGAC - GG  AGTTCAGGAGGAGCGTCAACCGG | D3S13  WT | 2/15 | heterozygous | 13.33% |
| # (2) | AGTTCAGGAGGAGCATCAACCGG  AGTTCAGGAGGAGCGTCAACCGG | +1  WT | 1/15 | heterozygous | 6.67% |
|  |  |  | 3/30 |  | 10% |

| **Table S5. Targeted genomic mutations of *FhNZZ* in T0 plants of *F. hindsii*.** #2-#47 are mutant plants for pK7WG2D:*FhNZZ*. The PAM is highlighted with a green background. Deleted nucleotides are highlighted in red. Inserted nucleotides are highlighted in blue. Replaced nucleotides are highlighted in green. plus (+) signs, letter D and letter S indicate the number of nucleotides inserted, deleted and replaced, respectively. The asterisks indicate the numbers of independent clones sequenced. | | | | | | | |
| --- | --- | --- | --- | --- | --- | --- | --- |
| *FhNZZ* | Target-1 | …316bp… | Target-2 | Mutation type | No. mutated /No. sequenced | genotype | mutation rate |
| WT | CCGCAGAGAGGAATGGGAGTGGC | …316bp… | CCATGGGGAGTCTTGCCTCAAA |  |  |  |  |
| #2 | CCGCAG - - - - - - - - - - - - - - - - -  CCGCAGAG - - - - - - - - - - - - - - -  CCGCAGAG - - - - - - - - - - - - - - -  CCGCAGGGAG - - - - - - - - - - - - -  CCGCAGAG - - GAATGGGAGTGGC  CCGCAGAAGAGGAATGGGAGTGGC  CCGCAGA - - - - - ATGGGAGTGGC  CCGCAGAAGAGGAATGGGAGTGGC  CCGCAG - GAGGAATGGGAGTGGC  CCGCAGAG - - GAATGGGAGTGGC  CCGCAGACCATGGCTGCTGGTGA | …316bp…  …316bp…  …673bp…  …316bp…  …633bp…  …633bp…  …633bp…  …307bp…  …316bp…  …316bp…  …316bp… | - - - - - - - GAGTCTTGCCTCAACA  - - - - - - - - - - -TCTTGCCTCAACA  - - - -AGAGAGTGAGGAAAGGAA  - - - - - - - GGAGTCTTGCCTCAACA  - - - - - - - - - -TTGAAAAAGTTGTTA  - - - - - - - - - -TTGAAAAAGTTGTTA  - - - - - - - - - -TTGAAAAAGTTGTTA  - - - - - - - - - - - - - - - - - - - - - - - - ACA  CCATGGGGGAGTCTTGCCTCAACA  CCATGGGGGAGTCTTGCCTCAACA  TCCTCTGGGAGTCTTGCCTCAACA | D340  D341  D673  D335S1  D635  +1D633  D638  +1D24  +1D1  +1D2  S338 | 2/13  1/13  1/13  1/13  1/13  2/13  1/13  1/13  1/13  1/13  1/13 | chimeric | 100% |
| #4 | CCGCAG - - - GGAATGGGAGTGGC  CCGCAGAGAGGAATGG - AGTG - C  CCGCAGAGAGGAATGG - AGTGGC  CCGCAGAGAGGAATGGGAGTGGC  CCGCAGAGAGGAATGGGAGTGGC  CCGCAGAGAGGAATGGAGG - - GC  CCGCAGAGAGGAATGGGAGTGGC | …316bp…  …316bp…  …316bp…  …316bp…  …316bp…  …316bp…  …316bp… | CCATGGGGAGTCTTGCCTCAACA  CCATGGGGAGTCTTGCCTCAACA  CCATGGGGAGTCTTGCCTCAACA  CCATGGGGGAGTCTTGCCTCAACA  CCATGGTGGAGTCTTGCCTCAACA  CCATGGGGAGTCTTGCCTCAACA  CCATGGGGAGTCTTGCCTCAACA | D3  D2  D1  +1  +1  D2S2  WT | 1/11  2/11  3/11  1/11  1/11  1/11  2/11 | chimeric | 81.8% |
| #7 | CCGCAGAGAGGAATGG - AGTG - C  CCGCAGAGAGGAATGG - AGTG - C  CCGCAGAGAGGAATGG - AGTGGC  CCGCAGAGAGGAATGGAGG - -GC  CCGCAGAGAGGAATGGGAGTGGC | …316bp…  …316bp…  …316bp…  …316bp…  …316bp… | CCATGGGGAGTCTTGCCTCAACA  CCATGGGGAGTCTTGCCTCAACA  CCATGGGGAGTCTTGCCTCAACA  CCATGGGGAGTCTTGCCTCAACA  CCATGGGGAGTCTTGCCTCAACA | D3  D2  D1  D2S1  WT | 1/11  2/11  1/11  1/11  6/11 | chimeric | 45.45% |
| #8 | CCGCAGA - - - - - - - - - - - - - - - -  - - - - - - - - - - - - ATGGGAGTGGC  CCGCAGAAGAGGAATGGGAGTGGC | …316bp…  …316bp…  …316bp… | - - - - - - - - AGTCTTGCCTCAACA  CCATGG - - - - TCTTGCCTCAACA  CCATGG - - - - TCTTGCCTCAACA | D340  D16  +1D4 | 5/8  1/8  2/8 | chimeric | 100% |
| #9 | CCGCAG - - - - - - - - - - - - - - - - -  CCGCAGAGAGGAATGG - AGTGGCT  CCGCAGAGAGGAATGGGAGTGGC  CCGCAGAGAGGAATGGGAGTGGC | ...316bp…  ...316bp…  ...316bp…  ...316bp… | - - - - - - - - - - - - - - - - -TCAACA  CCATGGGGAGTCTTGCCTCAACA  CCATGGGGGAGTCTTGCCTCAACA  CCATGGGGAGTCTTGCCTCAACA | D339  D4S2  +1  WT | 1/11  1/11  1/11  8/11 | chimeric | 27.27% |
| #13 | CCGCAGAGAGGAATGGGAGTGGC  CCGCAGAAGAGGAATGGAGTGGC  CCGCAGAGAGGAATGGGAGTGGC | ...316bp…  ...316bp…  ...316bp… | CCATGGGGAGTCTTGCCTCAACA  CCATGGGGAGTCTTGCCTCAACA  CCATGGGGAGTCTTGCCTCAACA | S1  S3  WT | 2/12  1/12  9/12 | chimeric | 25% |
| #15 | CCGCAGAGAAGGAATGGGAGTGGC  CCGCAGAGAGGGAATGGGAGTGGC  CCGCAGAGAGGAATGGGAGTGGC  CCGCAGAGAGGAATGGGAGTGGC | ...316bp…  ...316bp…  ...316bp…  ...316bp… | CCATGGGGAGTCTTGCCTCAACA  CCATGGGGAGTCTTGCCTCAACA  CCATGGGGGAGTCTTGCCTCAACA  CCATGGGGAGTCTTGCCTCAACA | +1  +1  +1  WT | 1/12  1/12  1/12  9/12 | chimeric | 25% |
| #21 | CCGCAG - - - - - - - - - - - - - - - - -  AAAGCGAATGCTCA - - - - -  CCGCAG - - - - - - - - - - - - - - - - - | …316bp…  …378bp…  …316bp… | - - - - - GGGAGTCTTGCCTCAACA  - - - - - - - - - - - - - - - - - - - - ACA  - - - - - - GGAGTCTTGCCTCAACA | D338  D378  D339 | 2/4  1/4  1/4 | chimeric | 100% |
| #24 | CCGCAG - - - - - - - - - - - - - - - - -  CCGCAGAG - - - - - - - - - - - - - - -  CCGCA - - - - - - - - - - - - - - - - - - | …316bp…  …316bp…  …316bp… | - - - - - - GGAGTCTTGCCTCAACA  - - - - - - - GAGTCTTGCCTCAACA  - - - - - - - GGGAGTCTTGCCTCAACA | D338  D338  D339 | 7/12  4/12  1/12 | chimeric | 100% |
| #25 | CCGCAG - - - - - - - - - - - - - - - - -  CCGCAG - - - GGAATGGGAGTGGC  CCCCAG - - - GGAAATGGAGTGGC | …316bp…  …316bp…  …316bp… | - - - - - - GGAGTCTTGCCTCAACA  CCATGGGGGAGTCTTGCCTCAACA  CCATGGGGGAGTCTTGCCTCAACA | D338  +1D3  +1D3S3 | 8/11  2/11  1/11 | chimeric | 100% |
| #27 | CCGCAG - - - - - - - - - - - - - - - - -  CCGCAGAAGAGGAATGGGAGTGGC  CCGCAGAGAGGAATGGGAGTGGC | …316bp…  ...316bp…  ...316bp… | - - - - - - GGAGTCTTGCCTCAACA  CCATGGGGAGTCTTGCCTCAACA  CCATGGGGAGTCTTGCCTCAACA | D339  +1  WT | 7/12  1/12  4/12 | chimeric | 66.67% |
| #28 | CCGCAG - - - - - - - - - - - - - - - - -  CCGCAGAG - - - - - - - - - - - - - - -  CCGCAGAG - - GAATGGGAGTGGC  CCGCAGAGA - - - ATGGGAGTGGC  CCGCAGAG - - GAATGGGAGTGGC  CCGCAGAG - - GAATGGGAGTGGC  CCGCAGAAGAGGAATGGGAGTGGC  CCGCAGA - - - - AATGGGAGTGGC | …316bp…  …316bp…  ...316bp…  ...316bp…  ...316bp…  ...316bp…  ...316bp…  ...316bp… | - - - - - - GGAGTCTTGCCTCAACA  - - - - - - - GAGTCTTGCCTCAACA  CCATGG - - - 23bp - - - TGATATTT  CCATGGTGGAGTCTTGCCTCAACA  CCATGGG - - - - - - TGCCTCAACA  CCATGG - GAGTCTTGCCTCAACA  CCATGGGGAGTCTTGCCTCAACA  CCATGG - GAGTCTTGCCTCAACA | D339  D338  D25  +1D3  D8  D3  +1  D5 | 3/10  1/10  1/10  1/10  1/10  1/10  1/10  1/10 | chimeric | 100% |
| #31 | CCGCAGA - - - - - ATGGGAGTGGC  CCGCAGAGAGGAATGGGAGTGGC | ...316bp…  ...316bp… | CCATGGGGGAGTCTTGCCTCAACA  CCATGGGGAGTCTTGCCTCAACA | +1D5  WT | 1/11  10/11 | heterozygous | 9.09% |
| #32 | CCGCAGAGAGGAATGGGAGTGGC  CCGCAGAGATGGAATGGGAGTGGC  CCGCAGAGAGGAATGGGAGTGGC | ...316bp…  ...316bp…  ...316bp… | CCATGGG - - - - - - - - CCTCAACA  CCATGGGGAGTCTTGCCTCAACA  CCATGGGGAGTCTTGCCTCAACA | D8  +1  WT | 4/9  1/9  4/9 | chimeric | 55.55% |
| #34 | CCGCAGA - - - - - ATGGGAGTGGC  CCGCAGAGGAGGAATGGGAGTGGC  CCGCAGAAGAGGAATGGGAGTGGC  CCGCAG - - - - GAATGGGAGTGGC  CCGCAGAG - - GAATGGGAGTGGC  CCGCAGAGAGGAATGGGAGTGGC | ...316bp…  ...316bp…  ...316bp…  ...316bp…  ...316bp…  ...316bp… | CCATGGGGGAGTCTTGCCTCAACA  CCATGGGGAGTCTTGCCTCAACA  CCATGG - GAGTCTTGCCTCAACA  CCATGGGGGAGTCTTGCCTCAACA  CCATGGGGGAGTCTTGCCTCAACA  CCATGGGGAGTCTTGCCTCAACA | +1D5  +1  +1D1  +1D4  +1D2  WT | 1/14  1/14  2/14  1/14  1/14  8/14 | chimeric | 42.86% |
| #36 | CCGCAGAGAGGAATGGGAGTGGC  CCGCAGAAGAGGAATGGGAGTGGC  CCGCAGAGGAGGAATGGGAGTGGC  CCGCAGAG - - GAATGGGAGTGGC  CCGCAGA - - - -AATGGGAGTGGC  CCGCAGAGAGGAATGGGAGTGGC | ...316bp…  ...316bp…  ...316bp…  ...316bp…  ...316bp…  ...316bp… | CCATGGGGGAGTCTTGCCTCAACA  CCATGGGGGAGTCTTGCCTCAACA  CCATGGGGAGTCTTGCCTCAACA  CCATGG - - - - TCTTGCCTCAACA  CCATGGGGAGTCTTGCCTCAACA  CCATGGGGAGTCTTGCCTCAACA | +1  +2  +1  D6  D4  WT | 1/12  1/12  1/12  1/12  1/12  7/12 | chimeric | 41.67% |
| #40 | CCGCAG - - - - - - - - - - - - - - - - -  CCGCAGAG - - - 33bp - - -  CCGCAG - - - - - - - - - - - - - - - - - | …316bp…  …298bp…  …316bp… | - - - - - - GGAGTCTTGCCTCAACA  C A - - - - - - - - - - TTGCCTCAACA  - - - - - - GGAGTCTTGCCTCAACA | D339  D43S1  D339 | 2/8  1/8  5/8 | chimeric | 100% |
| #43 | CCGCAGAGAGGAATGGGAGTGGC  CCGCAGAAGAGGAATGGGAGTGGC  CCGCAGAGAGGAATGGGAGTGGC | ...316bp…  ...316bp…  ...316bp… | CCATG - - - - - - - - - - - - - - - - - CA  CCATGGGGAGTCTTGCCTCAACA  CCATGGGGAGTCTTGCCTCAACA | D16  +1  WT | 1/13  1/13  11/13 | chimeric | 15.38% |
| #47 | CCGCAG - - - - - - - - - - - - - - - - -  - - - - - - - - - - - 56bp- - - - - - - - - - - - -  - - - - - - - - - - - 56bp- - - - - - - - - - - - - | …316bp…  ...316bp…  ...316bp… | - - - - - - GGAGTCTTGCCTCAACA  CCATGGGGGAGTCTTGCCTCAACA  CCATGG - - - - - - TTGCCTCAACA | D339  +1D56  D62 | 6/11  4/11  1/11 | chimeric | 100% |
|  |  |  |  |  | 127/205 |  | 62% |

| **Table S6. Raw statistics of the pedicel longitudinal diameter.** | | | | | | | | | | | | | | | | | |  |
| --- | --- | --- | --- | --- | --- | --- | --- | --- | --- | --- | --- | --- | --- | --- | --- | --- | --- | --- |
| Pedicel longitudinal diameter (mm) | | | | | | | | | | | | | | | | | |  |
| WT  (n=100) | 3.50 | 3.55 | 2.67 | 3.64 | 3.06 | 3.60 | 3.57 | 3.67 | 3.68 | 3.65 | 2.88 | 2.92 | 3.07 | 3.29 | 3.14 | 3.15 | 3.85 |  |
|  | 2.98 | 3.68 | 3.57 | 3.72 | 3.74 | 3.46 | 3.19 | 3.43 | 4.33 | 3.51 | 3.19 | 3.09 | 3.34 | 3.55 | 3.37 | 2.82 | 3.62 |  |
|  | 3.12 | 3.90 | 3.65 | 3.73 | 3.26 | 2.99 | 2.70 | 3.70 | 3.09 | 3.19 | 4.34 | 3.61 | 3.38 | 3.83 | 3.47 | 3.42 | 3.91 |  |
|  | 3.24 | 3.28 | 3.70 | 3.44 | 3.88 | 3.22 | 3.42 | 3.17 | 3.76 | 2.96 | 3.00 | 3.39 | 3.89 | 3.60 | 5.10 | 3.77 | 3.67 |  |
|  | 3.33 | 3.35 | 2.72 | 3.51 | 3.69 | 3.20 | 3.13 | 4.36 | 3.52 | 3.27 | 3.90 | 3.47 | 3.36 | 2.67 | 3.37 | 3.13 | 3.41 |  |
|  | 4.62 | 3.92 | 3.80 | 3.40 | 4.21 | 3.57 | 3.52 | 3.54 | 3.04 | 3.13 | 3.69 | 4.63 | 3.50 | 3.07 | 3.59 |  |  |  |
| D-CR#7  (n=100) | 7.45 | 4.76 | 6.35 | 5.86 | 6.72 | 5.98 | 6.69 | 4.75 | 6.31 | 5.18 | 7.06 | 5.86 | 5.81 | 5.99 | 6.28 | 6.12 | 5.98 |  |
|  | 5.52 | 6.71 | 5.52 | 6.57 | 5.79 | 5.27 | 5.85 | 5.27 | 4.75 | 5.63 | 6.35 | 6.81 | 5.51 | 6.10 | 5.86 | 6.02 | 5.29 |  |
|  | 5.14 | 6.05 | 4.61 | 5.90 | 6.08 | 5.89 | 6.29 | 6.03 | 5.56 | 5.89 | 4.58 | 6.12 | 6.28 | 5.67 | 6.55 | 5.36 | 6.12 |  |
|  | 5.56 | 6.32 | 6.60 | 6.95 | 6.64 | 5.31 | 5.76 | 7.69 | 6.05 | 6.54 | 4.71 | 5.09 | 5.27 | 5.18 | 6.16 | 5.19 | 4.76 |  |
|  | 7.03 | 6.96 | 5.45 | 5.49 | 6.83 | 5.05 | 5.08 | 6.67 | 6.40 | 6.67 | 6.22 | 7.41 | 5.25 | 4.40 | 6.05 | 6.18 | 5.19 |  |
|  | 5.95 | 5.24 | 6.07 | 4.48 | 6.31 | 4.59 | 5.22 | 6.16 | 6.24 | 5.40 | 5.46 | 5.12 | 6.72 | 5.15 | 5.87 |  |  |  |

| **Table S7. The primers used in the CRISPR experiment.** | |
| --- | --- |
| Primer name | Sequences (5’-3’) |
| 131-F | CAACCCTTGATCGGGGAAGAACAGT |
| 131-R | TAACTATCGTCTTGAGTCCAACCCG |
| 131-ccdb-F | GAGACGAGATCTAGTCTGAGTCGACTGGCCGATTCATTAATGCAGC |
| 131-ccdb-R | GCTCTAAAACAGAGACGGTCGACACCCGCCGCGCTTAATG |
| DUO1-F | ATGGAAGGAAAGAGAGAAGAGATAA |
| DUO1-R | TCACCAATCTGATGGACTCGGAA |
| DUO1-1CR-F | ATTGGTGGAGCGTTGTGGCGG |
| DUO1-1CR-R | ACACACAAACCCATCTCAAATTCGA |
| DUO1-2CR-F | ATTCCACTGTTGTAATCCGCTGTT |
| DUO1-2CR-R | ATCTGATGGACTCGGAAGTGGCTCG |
| NZZ-CR-F | AAAAATACAAAGCAACAATGTCCTG |
| NZZ-CR-R | CTAGCACCATAAACATTCATCCCT |
| DUO1- 1OFF-F | GTGTGTGTCGCTGATACTTGCTA |
| DUO1- 1OFF-R | TAAACACTTCCATCTATGTCAAACC |
| DUO1- 2OFF-F | ATGCCTTGCCACATTACTACTCA |
| DUO1- 2OFF-R | GACTCAATAGAAATCCTCTTTTCTCTTA |
| NZZ-1OFF1-F | TGACCAACAACGGACTTGTCAACC |
| NZZ-1OFF1-R | CCAAAACTCGCACAGGCAGAC |
| NZZ-1OFF2-F | TAGGGCTTCATTTCCTGAATGG |
| NZZ-1OFF2-R | CAAACTGCCAAAAGACGTGATC |
| NZZ-2OFF1-F | TCTATGTGCCTTTGCTTCTATCCC |
| NZZ-2OFF1-R | GGGACCGAGTCAAAAGGCAA |
| NZZ-2OFF2-F | AAGTGTTCTGTTTTGTCCAGGTTC |
| NZZ-2OFF2-R | CCAACAACCTGAACCATACATAGC |
| NZZ-2OFF3-F | CCTGGCTCTGCGTTCAATTTACTG |
| NZZ-2OFF3-R | GGTGCTGAGGGGTATATTCCGAAG |
| Cas9-F | CCGAGGGAATGAGAAAGC |
| Cas9-R | CGGTGATAGACTGGTGGATAAG |

| **Table S8. The primers used for expression analysis.** | |
| --- | --- |
| Primer name | Sequences (5’-3’) |
| GEX2-F | CAAGCTGGATGTTTGTGGATAAC |
| GEX2-R | GGAAAGGGCAGAGTGAGAAA |
| MGH3-F | GCTTATCTCGTGGGTCTGTTC |
| MGH3-R | TCCTTGGGCATAATGGTGAC |
| DAZ2-F | GCGGACAGTAAGGTGAAGTTAG |
| DAZ2-R | GTCAGCATCCACATCAGTAGTT |
| DAZ3-F | CCAAGCACTAGGTGGTCATAAA |
| DAZ3-R | AGCAGGCAGATTAAGGTCAAG |
| TIP5;1-F | TACTGGGTTGGTCCTTTGTTC |
| TIP5;1-R | CCATCAGAAACTCCTGCAATAGA |
| CYCB1;1-F | CTTCTTGATGGGATTGGAGTAGG |
| CYCB1;1-R | GAGCCTGAGCCTTTGGTTTA |
| CYCB1;2-F | CTACGCCTTATGTGTTCCTAGTT |
| CYCB1;2-R | ACAGTGGGATAATGGCTGATAC |
| CYCB1;3-F | GCCAGAAACCCTCTATCTTACC |
| CYCB1;3-R | CGCCCAAATCTCCTCATACTT |
| CsACTIN-F | ATCTGCTGGAAGGTGCTGAG |
| CsACTIN-R | CCAAGCAGCATGAAGATCAA |
